# Gene expression and genetic analysis reveal diverse causes of recessive self-compatibility in *Brassica napus* L.

**DOI:** 10.1186/1471-2164-15-1037

**Published:** 2014-11-28

**Authors:** Wen Zhai, Jianfeng Zhang, Yong Yang, Chaozhi Ma, Zhiquan Liu, Changbin Gao, Guilong Zhou, Jinxing Tu, Jinxiong Shen, Tingdong Fu

**Affiliations:** National Key Laboratory of Crop Genetic Improvement, National Center of Rapeseed Improvement in Wuhan, Huazhong Agricultural University, Wuhan, 430070 People’s Republic of China; Zhengzhou Tobacco Research Institute, Zhengzhou, 450001 People’s Republic of China

**Keywords:** *Brassica napus*, Self-incompatibility (SI), *S* locus genes, Gene expression, Genetic analysis

## Abstract

**Background:**

*Brassica napus* (AACC) is self-compatible, although its ancestor species *Brassica rapa* (AA) and *Brassica oleracea* (CC) are self-incompatible. Most *B.napus* accessions have dominant self-compatibility (SC) resulting from an insertion of 3.6 kb in the promoter region of *BnSCR-1* on the A genome, while recessive SC in *B.napus* has rarely been observed. Expression and cloning of *SRK* and *SCR* genes and genetic analysis were carried out to dissect bases of recessive SC in *B.napus*.

**Results:**

Eleven accessions were screened to identify stable recessive SC and had the *S* genotype *BnS-7* on the A genome and *BnS-6* on the C genome similarly to *BrS-29* and *BoS-15*, respectively. In eight SC accessions, *BnSCR-7* and *BnSCR-6* were nearly undetectable and harbored no structural mutations in the promoters, while *SRK* genes were expressed at normal levels and contained intact CDS, with the exception of *BnSRK-7* in line C32. *SRK* and *SCR* genes were expressed normally but their CDSs had no mutations in three SC accessions. In self-incompatible S-1300 and 11 F_1_ hybrids, *SRK* genes and *BnSCR-1300* transcripts were present at high levels, while expression of the *BnSCR-7* and *BnSCR-6* were absent. Plants of *S* genotype *S*_*1300*_*S*_*1300*_ were completely SI, while SI phenotypes of *S*_*BnS-7*_*S*_*BnS-7*_ and *S*_*1300*_*S*_*BnS-7*_ plants were segregated in BC_1_ and F_2_ populations.

**Conclusions:**

The recessive SC in eight accessions is caused by the loss of function of *BnSCR-7* and *BnSCR-6* in pollen. Translational repression contributes to the recessive SC in three accessions, whose *SRK* and *SCR* genes were expressed normally and had identical CDSs to *BrS-29 or BoS-15*. SI in 11 F_1_ hybrids relies on the expression of *BnSCR-1300* rather than *SRK* genes. Other factor(s) independent of the *S* locus are involved in recessive SC. Therefore, diverse causes underlie recessive SC in *B. napus*, yielding insight into these complex mechanisms.

**Electronic supplementary material:**

The online version of this article (doi:10.1186/1471-2164-15-1037) contains supplementary material, which is available to authorized users.

## Background

*Brassica napus* (AACC) is an amphidiploid species developed from *B. rapa* (AA) and *B. oleracea* (CC). *B. rapa* and *B. oleracea* are self-incompatible, but *B. napus* is self-compatible. Elucidating how self-incompatibility (SI) was lost and self-compatibility (SC) was acquired has profound consequences for understanding the origin of *B. napus* as well as trait changes during the evolutionary process of plant polyploidization.

Self-incompatibility in *Brassica* is controlled sporophytically by a single multi-allelic locus called *S* locus (i.e., pollen SI phenotype is determined by the diploid genotype of the pollen-producing parent) [1]. The *S* locus consists of at least two genes: the stigma determinant *S*-locus receptor kinase gene (*SRK*) [2] and the pollen determinant *S*-locus protein 11 gene (*SP11*)/*S*-locus cysteine-rich protein (*SCR*) gene [3, 4] (*SP11*, referred to as *SCR* hereafter). The *S* locus is also termed the ‘*S* haplotype’ because *S-*locus genes are transmitted to progeny as one unit [5]. The *SRK*-*SCR* interaction is haplotype-specific and only occurs between the receptor and ligand encoded in the same *S*-locus haplotype [6–8]. *S* haplotypes can be divided into two classes. Class-II haplotypes are generally recessive to class-I haplotypes in pollen, but they are co-dominant in the stigma [9]. More than 100 and 50 *S* haplotypes occur in *B. rapa* and *B. oleracea*, respectively [10, 11]; only three and four haplotypes, respectively, are class II [12].

Self-incompatible *B. napus* strains have been developed via introgression from *B. oleracea* and *B. rapa*[13, 14] or via the resynthesis of *B. napus* from *B. oleracea* and *B. rapa*[15]. Thus, natural *B. napus* is usually thought to have lost *S* haplotypes, resulting in SC during evolution. However, latent *S* alleles are widespread in cultivated SC *B. napus*[16], and *S* haplotypes are widely distributed in cultivated *B. napus* lines [17, 18]. The most predominant *S* genotype is class-I *S* haplotype *BnS-1* on the A genome (similar to *B. rapa S-47* (*BrS-47*)) and class-II *S* haplotype *BnS-6* on the C genome (similar to *B. oleracea S-15* (*BoS-15*)). An insertion of 3.6 kb in the promoter region of *BnSCR-1* previously resulted in no gene expression, but the non-functional class-I *SCR* on the A genome suppressed the expression of the recessive *BnSCR-6* on the C genome, resulting in SC [17, 19]. However, SC in *B. napus* with two class-II *S* haplotypes has rarely been observed.

The *B. napus* self-incompatible line S-1300 contains two class-II *S* haplotypes, *BnS-1300* on the A genome (similar to *BrS-60*) and *BnS-6* on the C genome [18, 20]. In S-1300, SI is recessive in most accessions but dominant in some genetic backgrounds [21]; SI is determined by *BnS-1300*[22]. The suppression of *BnSCR-1300* by the non-functional *BnS-1* in most lines with dominant SC explains their dominant SC. Accessions with recessive SC usually have only two class-II *S* haplotypes, one on the A genome (similar to *BrS-29*) and the other on the C genome (similar to *BoS-15*) [23]. Furthermore, one recessive gene previously co-segregated with the *S*-locus *SCR* gene and was reported to control recessive SC in Bing409 [23], while at least two genes controlled the recessive SC of 97Wen135 [24]. It is puzzling that Bing409 and 97Wen135 are self-compatible but can maintain the SI of S-1300.

To uncover the basis of recessive SC in natural *B. napus*, 11 *B. napus* accessions were screened to identify stable recessive SC and had the *S* genotype *BnS-7* on the A genome and *BnS-6* on the C genome in this study. Genetic analysis, gene expression, and gene cloning suggest that diverse causes underlie recessive SC in *B. napus*.

## Results

### Screening *B. napus*with recessive SC

Of the 30 F_1_ hybrids derived from crossing SI line S-1300 as a mother with 30 SC lines, 11 were stably SI, with an average SCI of <1 in both Wuhan and Lanzhou (Table 1). Thus, 11 male parents (B409, 1728, 614, 1745, C32, 1241, 326, 1638, 89008, 230, and 242) maintained the SI of S-1300 and displayed stable recessive SC.

**Table 1:** **Plants SI phenotype of 30 F**_**1**_ **hybrids developed from S-1300 as the mother line**

Male parent	Wuhan, 2010.5			Lanzhou, 2010.8			SI stability
	SI	PSI	SC	SI	PSI	SC	
128-2	0	1	5	0	1	3	
131-2	—	—	—	0	2	7	
173-1	2	3	2	6	2	2	
177-1	6	1	1	—	—	—	
182-1	0	3	4	4	0	0	
230-1	10	2	0	13	0	0	+
242-1	8	1	0	5	0	0	+
326-2	7	0	0	9	2	0	+
336-1	1	4	2	5	3	1	
360-2	2	5	0	9	0	0	
614-1	6	0	0	6	0	0	+
1100-1	0	1	6	5	0	0	
1122-1	4	3	0	1	2	4	
1241-1	13	1	0	7	0	0	+
1242-1	6	1	0	0	0	6	
1621-1	0	4	3	1	0	1	
1638-1	7	1	0	7	1	0	+
1728-1	7	0	0	8	1	0	+
1731-2	0	0	0	0	0	0	
1745-1	7	2	0	7	0	0	+
1756-1	7	0	0	—	—	—	
1760-2	7	1	0	2	4	2	
1771-1	1	2	4	7	1	0	
89008	8	1	0	10	0	0	+
68-1Chang	1	2	3	9	0	0	
B409	11	2	0	7	2	0	+
C32	12	2	0	6	0	0	+
D29	0	0	6	0	0	1	
ZY2045-2	4	0	0	—	—	—	
Huashuang5	5	2	0	2	5	3	

SI: self-incompatible, SCI <2; PSI (partially SI): 2 ≤ SCI <10; SC: self-compatible, SCI ≥10; +: stable recessive SC accessions. SCI was calculated as the number of seeds per flower.

### *S*haplotypes in recessive SC *B. napus*

**Figure 1:**
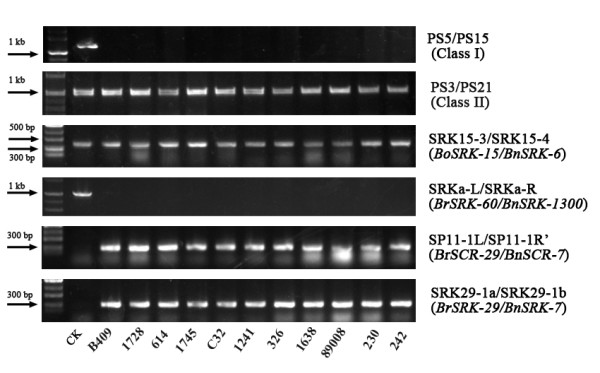
***S*** **haplotype identification.** Control (CK) is Westar in PS5/PS15 and S-1300 in the other five primer pairs.

The *S* haplotypes of the 11 accessions were identified with the primer combinations in Table 2. There was no amplification by the class-I specific primer pair PS5/PS15, but amplification did result from class-II specific primers PS3/PS21 (Figure 1), indicating that the 11 accessions only had class-II *S* haplotypes.

**Table 2:** **Primers for** ***S*** **haplotype identification**

Primer	Nucleotide Sequence 5′ to 3′	Length (bp)	***S*** haplotype	Reference
PS3	ATGAAAGGGGTACAGAACAT	1000	Class II	[25]
PS21	CTCAAGTCCCACTGCTGCGG			
PS5	ATGAAAGGCGTAAGAAAAACCTA	1340	Class I	[25]
PS15	ATGAAAGGCGTAAGAAAAACCTA			
SRK15-3	ATTCGATTGTGTTTCAGGCTC	380	*BoSRK-15*	[22]
SRK15-4	TCGACATGGTGATTTGGTTC			
SRKa-L	CAAGTTCTAATGAACGAGGTGG	1058	*BrSRK-60*	[20]
SRKa-R	CTGAGGAATAATAGGAGATACG			
SP11a-L	CAGAAGTCATGAGATATGCTAC	303	*BrSCR-29*	[23]
SP11a-R	ATTAGTAACATTCGGTCCG			
SRK29-1a	TATCATTAAGAATTCATCCGACCT	300	*BrSRK-29*	This study
SRK29-1b	TCATCGTCACGCCTAGAATAAG

Primer pairs SRK15-3/SRK15-4, SRKa-L/SRKa-R, and SP11a-L/SP11a-R were designed to amplify class-II *S*-locus genes *BoSRK-15*, *BrSRK-60*, and *BrSCR-29*, respectively. The 11 SC accessions and SI line S-1300 yielded a fragment with the same size (380-bp) which amplified by primer pair SRK15-3/SRK15-4 (Figure 1). This fragment was 100% identical to *BnSRK-6* (AB270772.1), indicating that the 11 accessions carried *BnS-6* (*BoS-15*) on the C genome.

Primer pair SRKa-L/SRKa-R produced a fragment of ~1000-bp only in S-1300, while SP11a-L/SP11a-R amplified a 303-bp DNA fragment in each of the 11 accessions (Figure 1). The 303-bp fragment was 100% identical to *BrSCR-29* (AB067449.1), which is named *BnS-7* in *B. napus*[17]. To confirm that the 11 SC accessions had *BnS-7* (*BrS-29)* on the A genome, primer pair SRK29-1a/SRK29-1b was designed based on a fragment deletion of *BnSRK-7* compared with sequences *BnSRK-6* and *BnSRK-1300*. The 11 SC accessions yielded identical 300-bp fragments, but S-1300 did not (Figure 1). This fragment was 100% identical to *BrSRK-29* (AB008191.1), confirming that *BnS-7* (*BrS-29*) was on the A genome.

### Gene expression of *SRK*and *SCR*

**Figure 2:**
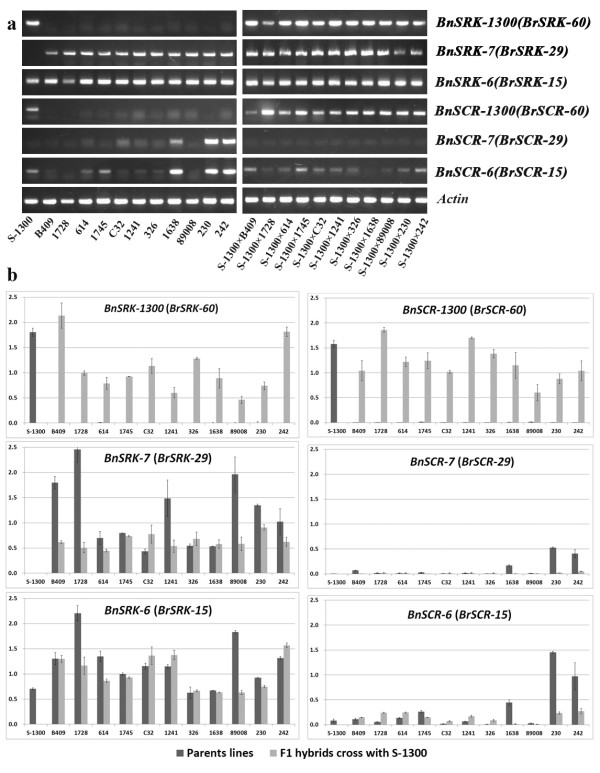
***SRK*** **and** ***SCR*** **expression in parental lines (S-1300 and 11 SC accessions) and F**_**1**_ **hybrids. (a)** Semi-quantitative real-time PCR **(b)** Quantitative real-time PCR, Error bars represent standard error (n = 3).

To detect relationships between *S*-locus genes expression and SI phenotype, specific primers based on *S*-locus genes were designed for qRT-PCR (Additional file 1: Table S1). Stigmas from parents and F_1_ hybrids, *BnSRK-7* and *BnSRK-6* expressed normally (Figure 2a) at a mean value of 1.05 (Figure 2b). Thus, *SRK* genes expression is co-dominant in stigma.

In anther of self-incompatible S-1300 and F_1_ hybrids, *BnSCR-1300* transcripts were present at high levels (>0.6), while *BnSCR-7* and/or *BnSCR-6* were nearly undetectable (Figure 2b). *BnSCR-7* was only expressed in anthers from three males (1638, 230, and 242) (Figure 2a), with expression values of 0.17, 0.52, and 0.41 (Figure 2b), respectively; no expression was detected in other males (Figure 2a). Similarly, *BnSCR-6* was expressed in 1638, 230, and 242 (Figure 2a), with values of 0.44, 1.45, and 0.97, respectively (Figure 2b), but little or no expression in other lines (<0.3) (Figure 2b). Thus, *BnSCR-1300* expression is necessary for the SI of S-1300 and F_1_ hybrids, and diverse expression patterns of the *SCR* genes occur across SC accessions.

### Cloning and sequence analysis of *S*-locus genes

**Figure 3:**
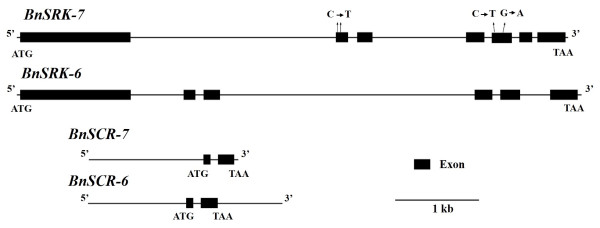
**Structure of** ***S*** **-locus genes and nucleotide sequence alignment.** Arrows sites show 4 base pairs diversity dispersed in exons 2 and 5 of C32.

Sequence mutations in the *BnS-6* and *BnS-7 S*-locus genes are thought to be responsible for recessive SC. Thus, 11 SC accessions were used to clone *SRK* from the A and C genomes. Primer combinations BnSRK7-2a/BnSRK7-2b (Additional file 2: Table S2) produced a 2590-bp sequence that contained the full *BnSRK-7* CDS. Sequence alignment showed that 10 SC accessions were 100% identical to *BrSRK-29* (AB008191.1), with four different base pairs in line C32 dispersed in exons 2 and 5 (Figure 3). Primer pair BnSRK6-1a/BnSRK6-1b produced a 2577-bp fragment with the full-length *BnSRK-6* CDS from each SC accession. These fragments shared 100% sequence identity with *BnSRK-6* (AB270772.1).

As only three male SC accessions (1638, 230, and 242) expressed *BnSCR-7* and *BnSCR-6* (Figure 2), cDNA from their anthers was used as template to clone the full CDS of *SCR.* Fragments 340-bp and 292-bp in length were obtained with primer pairs BnSCR7-2/BnSCR7-4 and BnSCR6-1a/BnSCR6-1b (Additional file 2: Table S2), respectively, with 100% identity to *BrSCR-29* (AB067449.1) and *BnSCR-6* (AB270774.1), respectively.

Primer combinations were developed to amplify genomic DNA including the 5′ promoter regions of *BnSCR-7* and *BnSCR-6*. Primer combination BnSCR7-3/BnSCR7-4 amplified a 1784-bp fragment of *BnSCR-7* that included 1367-bp 5′ upstream of the translation initiation site (Additional file 2: Table S2). All *BnSCR-7* sequences from the 11 SC accessions were 100% identical. Combination of the CDS and genomic DNA sequences revealed that *BnSCR-7* is 367-bp long and contains two exons and one intron.

Primer combinations BnSCR6-4a/BnSCR6-4b and BnSCR6-2a/BnSCR6-2b were developed to amplify 1193-bp and 1268-bp fragments of *BnSCR-6*, respectively. A 2320-bp fragment was obtained by combining the two fragments. This fragment encompasses 1176-bp of sequence 5′ upstream of the translation initiation site. All obtained *BnSCR-6* sequences were 100% identical. Combining the CDS and genomic DNA sequences indicated that *BnSCR-6* is 377-bp long, with two exons and one intron. Taken together, these observations show that the full genomic DNA sequences, including the 5′ promoter regions of *SCR*, have no sequence variation in 11 SC accessions, regardless of differences in expression.

### Genetic analysis of recessive SC

The F_2_ populations and two BC_1_ populations from six males were used for genetic analysis of recessive SC. Segregation of the SI/SC phenotype demonstrated that the SC of three males (1728, 614, and 1638) was controlled by a single locus (Table 3). However, *χ*^2^ values >3.84 were observed in populations (S-1300 × 326) × 326, (S-1300 × 230) × 230, (S-1300 × 89008) × 89008, and (S-1300 × 89008) F_2_, revealing that the SC of lines 326, 230, and 89008 was not controlled by a single locus. Separate analyses of four males (614, 326, 230, and 89008) conducted over two years returned similar results (Table 3).

**Table 3:** **Genotypes and phenotypes in segregating populations**

Population	Genotype ^a^			SI/SC phenotype	Expected ratio	***χ*** ^2^
	S _1300_	***S***_***BnS-7***_	S _1300_ ***S***_***BnS-7***_			
Year 2012						
(S-1300 × 1728) × S-1300	84/0^b^		78/0	162/0	1:0	0
(S-1300 × 1728) × 1728		11/28	27/9	38/37	1:1	0.03
(S-1300 × 1728) F_2_	33/0	12/26	48/7	93/33	3:1	0.1
(S-1300 × 614) × S-1300	111/0		105/0	216/0	1:0	0
(S-1300 × 614) × 614		9/69	65/15	74/84	1:1	0.64
(S-1300 × 614) F_2_	35/0	9/31	71/12	115/43	3:1	0.36
(S-1300 × 1638) × S-1300	56/0		55/0	111/0	1:0	0
(S-1300 × 1638) × 1638		9/49	54/12	63/61	1:1	0.04
(S-1300 × 1638) F_2_	37/0	14/27	67/8	118/35	3:1	0.44
(S-1300 × 326) × S-1300	73/0		76/0	149/0	1:0	0
(S-1300 × 326) × 326		93/172	232/22	325/194	1:1	32.56
(S-1300 × 326) F_2_	122/0	24/93	196/37	342/130	3:1	1.56
(S-1300 × 230) × S-1300	42/0		38/0	80/0	1:0	0
(S-1300 × 230) × 230		8/48	62/0	70/48	1:1	3.74
(S-1300 × 230) F_2_	54/0	7/38	85/0	146/38	3:1	1.63
(S-1300 × 89008) × S-1300	43/0		36/0	79/0	1:0	0
(S-1300 × 89008) × 89008		11/28	31/0	42/28	1:1	2.41
(S-1300 × 89008) F_2_	42/0	16/33	87/0	145/33	3:1	4.57
Year 2013						
(S-1300 × 614) × S-1300	57/0		64/0	121/0	1:0	0
(S-1300 × 614) × 614		13/33	40/12	53/45	1:1	0.66
(S-1300 × 614) F_2_	33/0	11/23	72/15	116/38	3:1	0.03
(S-1300 × 326) × S-1300	61/0		57/0	118/0	1:0	0
(S-1300 × 326) × 326		19/40	53/15	72/55	1:1	2.02
(S-1300 × 326) F_2_	33/0	15/24	55/12	103/36	3:1	0.35
(S-1300 × 230) × S-1300	112/0		124/0	236/0	1:0	0
(S-1300 × 230) × 230		16/35	49/0	65/35	1:1	8.41
(S-1300 × 230) F_2_	21/0	5/23	54/0	80/23	3:1	0.15
(S-1300 × 89008) × S-1300	30/0	0	25/0	55/0	1:0	0
(S-1300 × 98008) × 89008	0	7/13	21/0	28/13	1:1	4.78
(S-1300 × 89008) F_2_	34/0	14/21	71/0	119/21	3:1	6.94

SI: self-incompatible, SCI <2; SC: self-compatible, SCI ≥2.

^a^Primer combinations SRKa-L/SRKa-R and SRK29-1a/SRK29-1b were used to detect *BnS-1300* and *BnS-7*, respectively.

^b^Number of investigated plants with the SI/SC phenotype.

Primer combinations SRKa-L/SRKa-R and SRK29-1a/SRK29-1b (Table 2), were used to determine the inheritance of *S*_*1300*_ and *S*_*BnS-7*_ and to clarify the role of *S* genotypes in determining the SI phenotype. All *S*_*1300*_*S*_*1300*_ plants were completely SI in all progeny from six accessions; all *S*_*1300*_*S*_*BnS-7*_ plants were SI in progenies from lines 230 and 89008. However, SI plants of genotype *S*_*BnS-7*_*S*_*BnS-7*_ were observed in all populations, and some plants with genotype *S*_*1300*_*S*_*BnS-7*_ were SC in a population descended from four males (1728, 614, 1638, and 326) (Table 3). These results suggest that factor(s) independent of the *S* locus are involved in recessive SC, but that the *S* haplotype *BnS-1300* in the A genome is necessary for SI.

## Discussion

### Recessive SC in *B. napus*has diverse causes

SI has been used for hybrid breeding in *B. rapa* and *B. oleracea*. As *B. napus* is an oil crop, its hybrids should be fertile for harvesting seeds. On the other hand, a SI line must be propagated on a large scale to produce many hybrid seeds. The SI of line S-1300 is recessive in most accessions but dominant in some genetic backgrounds [21]. Therefore, it has been utilized for three-component hybrid breeding via SI F_1_ hybrids [21, 26]. Recessive SC lines thus play a key role in hybrid breeding.

Here, 11 accessions were screened to identify stable recessive SC and had the *S* genotype *BnS-7*/*BnS-6*. In 8/11 accessions, expression of *BnSCR-7* and *BnSCR-6* was nearly absent, but *SRK* genes were expressed at normal levels (Figure 2). *SRK* genes in these accessions contained no CDS mutations, with the exception of 4-bp in *BnSRK-7* in line C32 (Figure 3). These data indicate that SC is caused by the loss of function of *BnSCR-7* and *BnSCR-6* in the pollen of these eight accessions. However, *BnSCR-7* and *BnSCR-6* harbor no structural mutations in the promoters of these lines, rendering the mechanism of this loss of function unclear.

Sequence insertion/deletion causing loss-function of *SCR* was previously reported to cause SC. The SC *B. rapa* cultivar Yellow Sarson, which has a self-compatible class-I *S* haplotype (*S-f2*), contains an 89-bp deletion in the *SCR* promoter region; this deletion resulted in the production of no transcript, which caused the loss of function in the *S-f2* homozygote. The expression of recessive class-II *SCR-60* was suppressed in the *S-f2/S-60* heterozygote by non-functional class-I *SCR-f2*[27]. No expression of the class-I dominant *SCR* on the A genome resulted from the insertion of ~3.6 kb in the promoter region; this insertion’s suppression of the recessive locus *BnSCR-6* on the C genome explains the SC of *B. napus* accessions with the *S* genotype of *BnS-1/BnS-6*[17, 19]. SC in *A. thaliana* is also caused by a 213-bp inversion in the male-specific gene *SCR* that inhibits the transcription of *SCR*[28]. However, we were surprised that *SRK* and *SCR* were expressed normally in three SC accessions in the present investigation (Figure 2). The *SCR* and *SRK* CDSs in these lines had no mutations relative to *BrS-29* and *BoS-15*, implying that other factors contribute to SC by taking part in translational repression. Therefore, diverse causes result in recessive SC in *B. napus*.

### Factor(s) independent of the *S*locus contribute to SC in *B. napus*

Other factor(s) independent of the *S* locus may control recessive SC, based on our observations that *S*_*1300*_*S*_*1300*_ plants are completely SI, while *S*_*BnS-7*_*S*_*BnS-7*_ and *S*_*1300*_*S*_*BnS-7*_ plants segregated SI phenotypes in their progeny (Table 3). These observations are consistent with those of Ekuere et al. [16], who identified a latent *S* allele in at least two oilseed rape cultivars; the *S* phenotype of these latent alleles was masked by a suppressor system common to oilseed rape. A modifier was also proposed to cause transient SI in *A. thaliana*[29, 30]. Liu et al. [31] demonstrated that transient SI is caused by a hypomorphic allele of *PUB8* that regulates *SRK* transcript levels, and suggested that disruption or down-regulation of the *S*-locus recognition genes was a major mechanism for the switch to self-fertility in *A. thaliana.* Although genetic analyses are not completely consistent with our previous results [23, 24], SI plants with genotype *S*_*BnS-7*_*S*_*BnS-7*_ and SC plants with genotype *S*_*1300*_*S*_*BnS-7*_ are useful for mapping and characterizing the other factor(s) or suppressor(s) in this system.

In the Brassicaceae, self-recognition involves *SRK*-*SCR* interaction and signal transmission. Any factor that suppresses mRNA expression of *SRK* or *SCR* or disrupts subsequent signal transduction would cause the breakdown of SI. Several proteins have been shown to affect SI response in *Brassica*, such as the armadillo repeat-containing protein ARC1 [32], the thioredoxin h-like proteins THL1 and THL2 [33], and M-locus protein kinase MLPK [34]. However, MLPK, ARC1, and Exo70A1 orthologs do not contribute to the SI response in *A. thaliana SRK-SCR* transformants [35]. The *ARC1*-related U-box gene *AtPUB2*, which is highly expressed in the stigma, does not function in SI either [36]. Further investigations will be needed to determine whether the factor(s) proposed in the present study consist of these proteins.

### Dominant/recessive relationships in recessive class-II *S*haplotypes

In general, *S* haplotypes in *Brassica* exhibit dominant/recessive relationships in pollen and co-dominant relationships in stigma [9]. Some *S* haplotypes are hierarchically dominant; in *B. rapa*, the order is *S9* > *S44* > *S60* > *S40* > *S29*[37]. Of these *S* haplotypes, *S44*, *S60*, *S40* are dominant in some cases but recessive in others, based on the expression level of *SCR*[38]. The SI line S-1300 and 11 recessive SC accessions have a common *S* haplotype (*BnS-6*) on the C genome, but different *S* haplotypes on the A genome (*BnS-1300* in S-1300 and *BnS-7* in the 11 accessions). *BnSCR-1300* (similar to *BrSCR-60*) was only expressed in S-1300 and the 11 F_1_ hybrids, while *BnSCR-7* (similar to *BrSCR-29*) and *BnSCR-6* (similar to *BoSCR-15*) were nearly undetectable (Figure 2). In pollen, *BrSCR-60* is dominant over *BrSCR-29*[39]. The SI of F_1_ hybrids may be due to the dominant, functional *BnSCR-1300* on the A genome, to which *BnSCR-7* is recessive.

Dominant/recessive relationships between class-I and class-II *S* haplotypes are regulated by DNA methylation of the promoter of the recessive *SCR* gene [40]; this methylation is triggered by a *trans*-acting small non-coding RNA [41]. However, the mechanism underlying the dominant/recessive relationships between two class-II *S* haplotypes has not been reported to date. Previously, *BoSCR-15* was 95.5% identical at the amino-acid level to *BrSCR-60,* but only 57.6% identical to *BrSCR-29*[12]. If the dominant/recessive relationship between two *S* haplotypes in *B. rapa* and *B. oleracea* is present in *B. napus*, as observed by Okamoto et al. [17], then *BnSCR-1300* and *BnSCR-6* may be co-dominant, with both dominant to *BnSCR-7*. However, we did not detect *BnSCR-6* transcripts in the SI line S-1300, and neither *BnSCR-6* nor *BnSCR-7* was expressed in the anthers of eight SC accessions; the other three SC accessions clearly expressed *BnSCR-6* and *BnSCR-7* (Figure 2). Our observations cannot be explained by any dominant/recessive relationship among the class-II *BnSCR-1300*, *BnSCR-6*, and *BnSCR-7*. The SI line S-1300 and the recessive SC accessions employed here are unique materials for dissecting the dominant/recessive relationship and its mechanisms in class-II *S* haplotypes.

Much progress has been made toward elucidating the mechanism of SC in *B. napus*, but many questions persist, such as the roles of *SRK* and *SCR* in self-recognition, the dominant/recessive relationships between recessive class-II *S* haplotypes, and the identities and functions of other factors involved in SI singling. Our study provides insight into the complex mechanisms of SC in *B. napus*, laying the groundwork to characterize the novel factor(s) affecting *S*-locus gene expression and SI signaling. Dissecting these pathways will help elucidate the mechanisms of recessive SC and further our understanding of the evolution of plants from diploid to autoploid species and the changes in self-fertility during polyploidization.

## Conclusion

The recessive self-compatible accessions screened in this study had two common class-II *S* haplotypes: *BnS-6* on the C genome and *BnS-7* on the A genome. Our observations of different *BnSCR-6* and *BnSCR-7* expression patterns across SC accessions, the reliance of SI on the expression of *BnSCR-1300* rather than *SRK* genes, and the contributions to SI phenotypes of factor(s) independent of the *S* locus according to the inheritance of segregating populations suggest that diverse causes underlie recessive SC in *B. napus*, yielding insight into these complex mechanisms and laying the groundwork to characterize the novel factor(s) affecting *S*-locus gene expression and SI signaling.

## Methods

### Plant material

Self-incompatible line S-1300 and 30 cultivated self-compatible *B. napus* accessions used in this study are highly inbred lines (Table 1). Line S-1300 contains low erucic acid and low glucosinolates and is derived from the double-high SI line 271, which was bred by introgressing an *S* haplotype of *B. rapa* Xishuibai into a *B. napus* line through interspecific hybridization [42, 43]. These lines are Chinese semi-winter types and are conserved in Huazhong Agricultural University, Wuhan, China.

Line S-1300 was crossed as a female with the self-compatible *B. napus* accessions to obtain F_1_ hybrids in March 2009, in Wuhan (located on the central of China). F_1_ hybrids were artificially bud-pollinated to produce F_2_ populations and separately backcrossed with the female and males to generate BC_1_ populations in March 2011 in Wuhan. Phenotypes of F_1_ hybrids were investigated in two natural environments: in May 2010 in Wuhan and in August 2010 in Lanzhou (located on the northwest of China). Because of large and hard work of investigating SI phenotype, F_2_ and BC_1_ populations deriving only from 6 males were randomly selected and investigated in May of 2012. In 2013 in Wuhan, 3 males whose recessive SC controlled by at least two loci repeated separate analyses, and one male whose recessive SC controlled by one locus was used as control, as our original focus was on the modifier might existed in the recessive SC accessions.

### Determination of SC index (SCI) and SI phenotype

SCI and SI phenotypes were determined using the methods previously described [20]: When 3–5 flowers were present on the major inflorescence of each plant, the major inflorescence and 2–3 branches were bagged. The bags were slapped gently every two days to ensure self-pollination and were removed two weeks later to allow natural seed development. After the seed pods matured, the seeds and flowers were counted, and the SCI was calculated as the number of seeds divided by the number of flowers. Approximately 100–150 flowers from each plant were investigated. SI phenotype of each plant were categorized as SCI <2 (SI), 2 ≤ SCI <10 (partially SI) and SCI ≥10 (SC).

### DNA isolation and PCR

Genomic DNA of each plant from SI line S-1300, 11 SC accessions, 11 SI F_1_ hybrids and 4552 individuals of F_2_ and BC_1_ populations, was isolated from young leaves using cetyltrimethyl ammonium bromide [44]. DNA concentration was measured using a Beckman spectrophotometer. DNA from three individuals in each of the SI line S-1300, 11 SC accessions and 11 SI F_1_ hybrids was mixed for PCR analysis. PCR was performed on a thermocycler (Model PTC-225, MJ Research) in a volume of 20 μL including 50 ng DNA template, 0.2 mM dNTP mix (Sangon, China), 0.5 μM of each primer, 1 U Taq DNA polymerase (MBI Fermentas, USA), 2.0 mM MgCl_2_, and 2 μL 10× Taq buffer. The PCR mixture was covered with 20 μL mineral oil [18]. PCR products were separated on a 1.0% agarose gel in 1× TAE buffer and detected by staining with ethidium bromide.

### RNA analysis

Thirty stigmas or anthers of mature buds from each of the SI S-1300 line and 11 F_1_ hybrids and the 11 SC accessions were taken into a 2.0 ml tube placing on a box filled with liquid nitrogen gas. Total RNA was extracted using TRIzol reagent (Invitrogen, Carlsbad, USA). Reverse transcription (RevertAid™ First Strand cDNA Synthesis Kit, Fermentas, USA) was carried out according to the manufacturer’s instructions. The first-strand cDNA mixture was diluted 10-fold with sterile distilled water and used as a template to amplify cDNAs of *SRK* or *SCR* for semi-quantitative real-time PCR. Quantitative real-time PCR was also performed on a Bio-Rad CFX-96 with SYBR Green (Bio-Rad, USA). Actin (GeneBank accession number: AF111812) was amplified and used as a positive control. PCR was performed under the following conditions: 95°C for 3 min, followed by 47 cycles of 95°C for 10 s, 60°C for 15 s, and 72°C for 30 s. Relative transcript levels were determined by the comparative 2^-ΔΔC^T method [45] in triplicate. All primers are listed in Additional file 1: Table S1. Primers were designed with Primer Premier 5.0 (http://www.PremierBiosoft.com) and synthesized by Invitrogen, Carlsbad, USA.

### Cloning and sequence analysis

A homologous candidate gene approach was used to generate the full coding DNA sequences (CDSs) of *SRK* and full sequences with the 5′ promoter regions of *SCR* in 11 SC *B. napus* accessions based on the CDSs of *BnS-7* (*BrS-29*) on the A genome and *BnS-6* (*BoS-15*) on the C genome. As *B. rapa S-60*, *B. rapa S-29*, and *B. oleracea S-15* are class-II *S* haplotypes with high sequence similarity, their sequence differences were taken into consideration when designing primers (Additional file 2: Table S2).

DNA fragments were excised from a 1.0% agarose gel, purified using the UNIQ-10 column Gel Recovery Kit (Sangon, China), and ligated into vector PMD18-T (Takara, Japan). Positive transformed clones were screened by PCR with M13-specific primers. Three positive clones from each ligation were sequenced with an ABI 3730 automatic sequencer (Sangon, China). Sequence analysis was performed using BLAST [46], ClustalX 2.0 [47], and DNASTAR (Windows version 5.0.2, DNASTAR, Madison, WI, USA).

## Electronic supplementary material

Additional file 1: Table S1: Primers used to analyze the expression of *SRK* and *SCR.* (XLSX 10 KB)

Additional file 2: Table S2: Primers for cloning *SRK* and *SCR*. (XLSX 10 KB)
